# Case report: A novel 5'-UTR-exon1-intron1 deletion in MLYCD in an IVF child with malonyl coenzyme A decarboxylase deficiency and literature review

**DOI:** 10.3389/fmed.2023.1160879

**Published:** 2023-05-03

**Authors:** Fang Xu, Yangyang Wu, Jiyi Huang, Yunguo Zhou, Fei Xu, Junkai Duan, Hong Li

**Affiliations:** ^1^Cardiology Treatment Center, Jiangxi Provincial Children's Hospital, Nanchang, China; ^2^JXHC Key Laboratory of Children's Cardiovascular Diseases, Jiangxi Provincial Children's Hospital, Nanchang, China; ^3^Pediatric Medical Department, Nanchang University, Nanchang, China

**Keywords:** MLYCD, malonyl coenzyme A decarboxylase deficiency, cardiomyopathy, novel mutation, metabolic disease

## Abstract

The subject of the study is an 11-month old IVF baby girl with the typical clinical manifestation of malonyl coenzyme A decarboxylase deficiency, including developmental delay, limb weakness, cardiomyopathy, and excessive excretion of malonic acid and methylmalonic acid. Whole genome sequencing (WGS) revealed a novel heterozygous nonsense mutation (c.672delG, p.Trp224Ter) in the MLYCD gene of the proband and her father and a novel heterozygous deletion in 5'-UTR-exon1-intron1 of the MLYCD gene of the proband and her mother. The patient's cardiac function and limb weakness improved considerably after 3 months of a low-fat diet supplemented with L-carnitine. Furthermore, mapping of gene mutations and clinical manifestations was done by case collection.

## Introduction

Malonyl-CoA decarboxylase (MCD, E.C.4.1.1.9) activity deficiency caused by MLYCD gene mutation contributes to Malonic aciduria (OMIM 248360) (1). When MCD activity is affected, malonyl coenzyme A accumulates in the cytoplasm and strongly and effectively inhibits carnitine palmitoyltransferase (CPT1) in the outer mitochondrial membrane, resulting in the inability of long-chain fatty acids to enter the mitochondrial matrix for further β-oxidation and energy supply (2). Elevation of plasma malonylcarnitine (C3DC) level and urinary malonate is unique to this autosomal recessive disorder. After the report of the first case in 1984 (3), 55 cases have been reported worldwide, among which three were Chinese. In this study, we reported a case of MCD deficiency caused by novel compound heterozygous mutations at MLYCD gene loci, expanding the spectrum of genetic variants in this disease.

## Methods

All cases in the literature review, except for one new case reported in this article, were searched in PubMed and CNKI database by the search term “(Malonyl Coenzyme A Decarboxylase Deficiency) OR (Malonyl-CoA Decarboxylase Deficiency)”, and 55 cases from 30 papers were collected. Information on clinical presentation as well as gene mutation was counted from the 30 retrieved papers and one case was excluded from the study for lacking clinical information.

### Ethical compliance

The study was approved by the Institutional Review Ethics Board of Jiangxi Provincial Children's Hospital (JXSETYY-YXKY−20220277). Written informed consent for molecular tests and publication was obtained from the patient's parents.

## Result

### Clinical report

The 11-month-old patient is the second child of a healthy non-consanguineous Chinese couple, with a birth weight of 2.9 kg. Both the proband and her healthy older sister are IVF babies. No obvious abnormality could be observed in the patient. Biochemical metabolites test performed on postnatal Day 3 disclosed that C3DC+C4OH in whole blood and glutaric acid, 3-hydroxyglutaric acid, and methylmalonic acid in urine were all upregulated. This subject was suspected of having MCD deficiency according to the marked elevation of malonic acid. Whole-exome sequencing (WES) revealed a novel paternally inherited heterozygous mutation (p. Trp224Ter) in the MLYCD gene (NM_012213.2). No further treatment was given to this child. During the following months, symptoms including developmental delay, weakness of limbs, and inability to stand with support gradually arose in the baby, and she was hospitalized in the Cardiac Center owing to developmental delay, weakness of limbs, and poor cardiac function.

Physical examination showed that the patient experienced a relatively delayed physical development, weakened muscle strength, decreased muscle tone in the four extremities, and weakened knee and Achilles' tendon reflexes.

### Laboratory tests

Urinary gas chromatography-mass spectrometry (GC/MS) showed significantly elevated levels of malonic acid (195.54 mmol/mol creatinine; reference 0–0.1 mmol/mol creatinine) and methylmalonic acid (66.25 mmol/mol creatinine; reference 0.2–3.6 mmol/mol creatinine). Slightly increased blood malonylcarnitine (0.54 uM; reference 0.03–0.31uM) was detected by tandem mass spectrometry (MS/MS). Biochemical test results indicated increased levels of B-type brain natriuretic peptide (530 pg/ml; reference 0–125 pg/ml) and ultrasensitive troponin (0.026 ng/ml, reference 0–0.012 ng/ml). Additional tests, including complete blood count, urinalysis, blood ammonia, and other blood biochemical indexes were all in the normal range.

#### Magnetic resonance imaging of the brain

Magnetic resonance imaging of the brain showed slightly broadened bilateral frontal space and widening and deepening of the sulcus.

#### Echocardiogram

Echocardiogram showed significant enlargement of the left ventricle accompanied by the low systolic function of the left heart with an ejection fraction of 41% and fractional shortening of 20.2% (Supplementary Figure 1 in Supporting Information).

#### Computerized tomography

Computerized tomography revealed an enlarged left ventricle and slight thickening of the left ventricular septal bundle and wall bundle.

No abnormality in 16-lead EEG and EMG was observed.

#### Denver developmental screening

Denver developmental screening implies the patient experiences delayed development in five developmental domains, including personal-social, gross-motor, fine-motor, language, and perceptive-cognitive.

### Genetic testing

**Figure 1:**
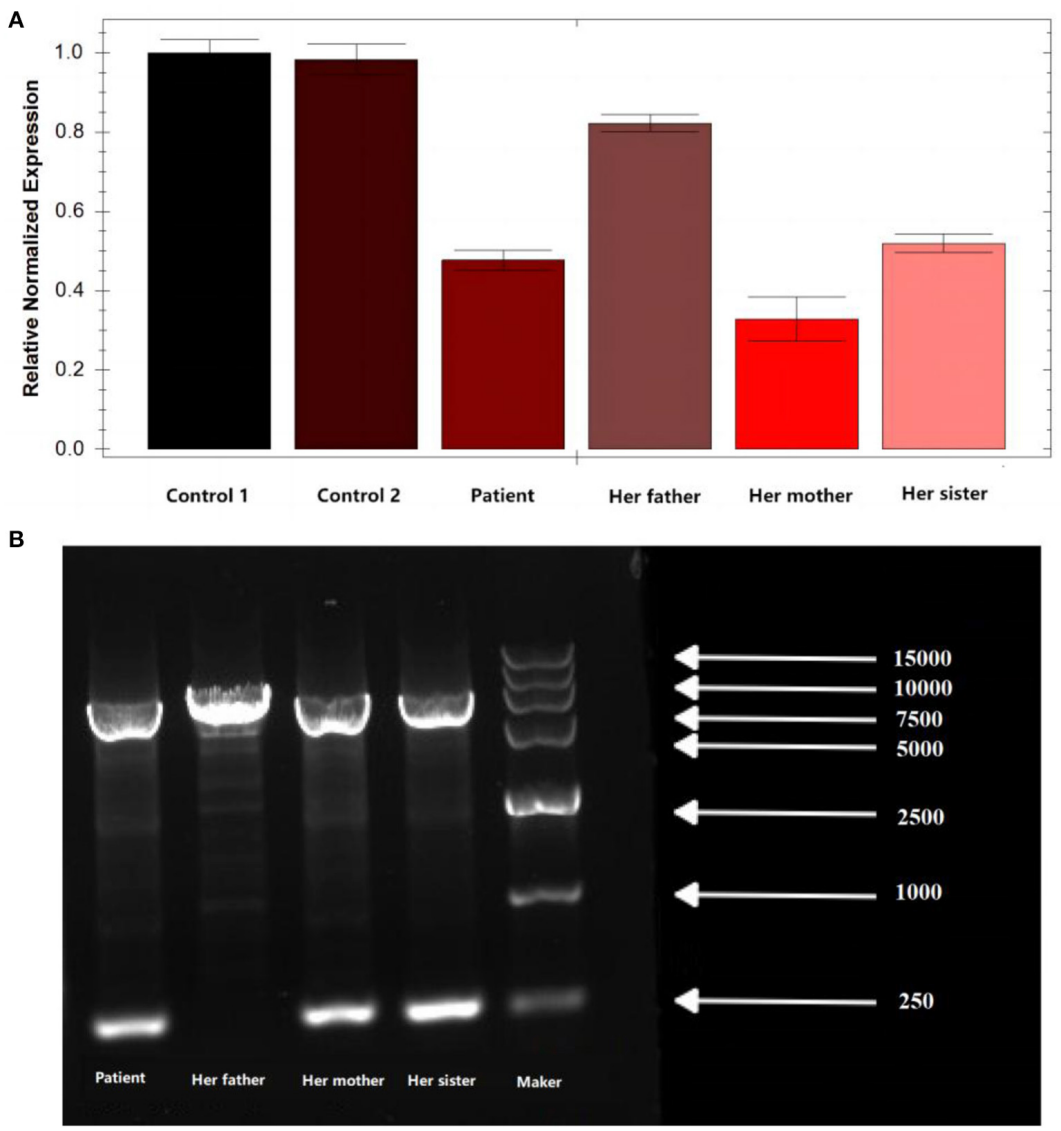
qPCR and agarose electrophoresis results from the patient's family. **(A)** qPCR results from the patient's family. **(B)** Results of agarose electrophoresis of the qPCR product from the patient's family. Her father has only one chromosome band with a fragment length of 7,181, which is the full length of the amplification product. In addition to this, the patient, her mother, and her sister have an additional chromosome band with a fragment length of 250 bp, which is the length of the truncated fragment.

**Figure 2:**
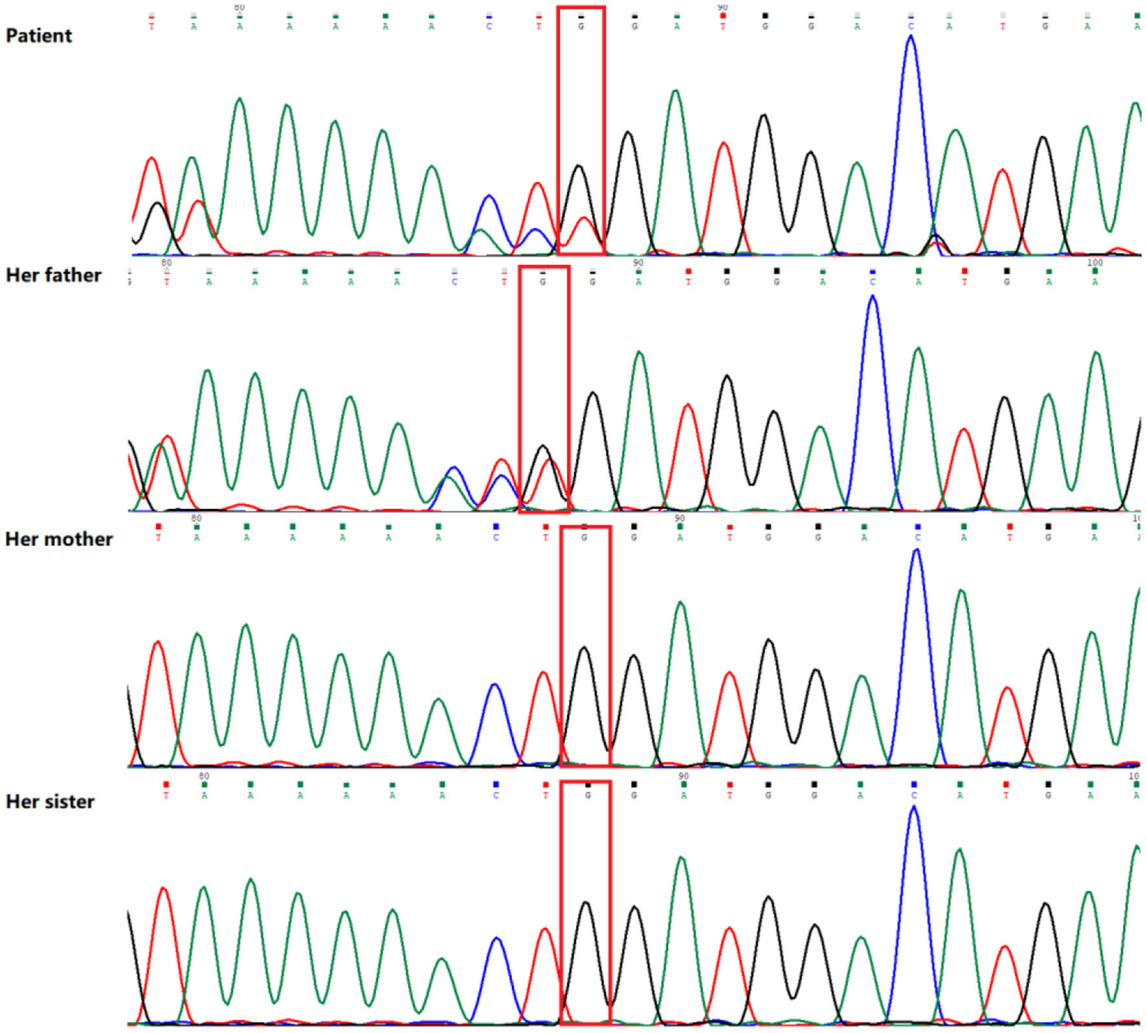
Sequence analysis results of the MLYCD gene of the patient and her family. The patient and her father have a novel heterozygous mutation (red box) in exon 3 (c.672del/p.Trp224*).

Peripheral blood samples were collected from the patient and the patient's parents and sister, followed by genomic DNA extraction. Whole genome sequencing (WGS) was conducted to examine the MLYCD gene, and a novel heterozygous mutation 5'-UTR-exon1-intron1 deletion was identified which her mother and sister also preserved. In addition, the copy number of MLYCD was estimated by quantitative PCR (qPCR), and the normal reference value is between 0.7 and 1.3. The test values for the patient, her mother, her sister, and her father were 0.47712, 0.32836, 0.51949, and 0.82228, respectively, indicating that the first three lost one copy of the MLYCD gene (Figure 1A). QPCR products of the three individuals were examined by agarose electrophoresis and all were found to have a truncated fragment of 250 bp (Figure 1B). Another mutation c.672delG in the patient and father, previously identified by WES, was also detected by WGS and confirmed by Sanger sequencing (Figure 2). Thus, the observed MCD deficiency was proved to be driven by a compound heterozygous pathogenic mutation comprised of the c.672delG mutation and the 5'-UTR-exon1-intron1 deletion in *MLYCD*. Both sites are not in the HGMD and gnomAD databases.

### Treatment

After admission, the child was given a low-fat-high-carbohydrate, high-MCT formula diet. Levocarnitine was commenced at a dose of 100 mg/kg/day. Anti-cardiac failure therapy comprised of digoxin, diuretics, and enalapril maleate was initiated. Anti-symptomatic measures such as taking sodium creatine phosphate and vitamin C to nourish the myocardium were also applied. Eight days after treatment, muscle tension and tone showed improvement. Additional echocardiography showed the heart size recovered slightly and left ventricular systolic function becomes slightly better than at admission. On day 9 after admission, the child was discharged and the original treatment protocol was continued. After 3 months of follow-up, her heart function has improved to a normal level. At the end of the 6-month treatment, digoxin was discontinued. The subject's growth and development were close to a normal level. She is now able to stand and walk with support. The results of the follow-up cardiac ultrasound are presented in Supplementary Table 1 in Supporting Information.

## Literature review

**Figure 3:**
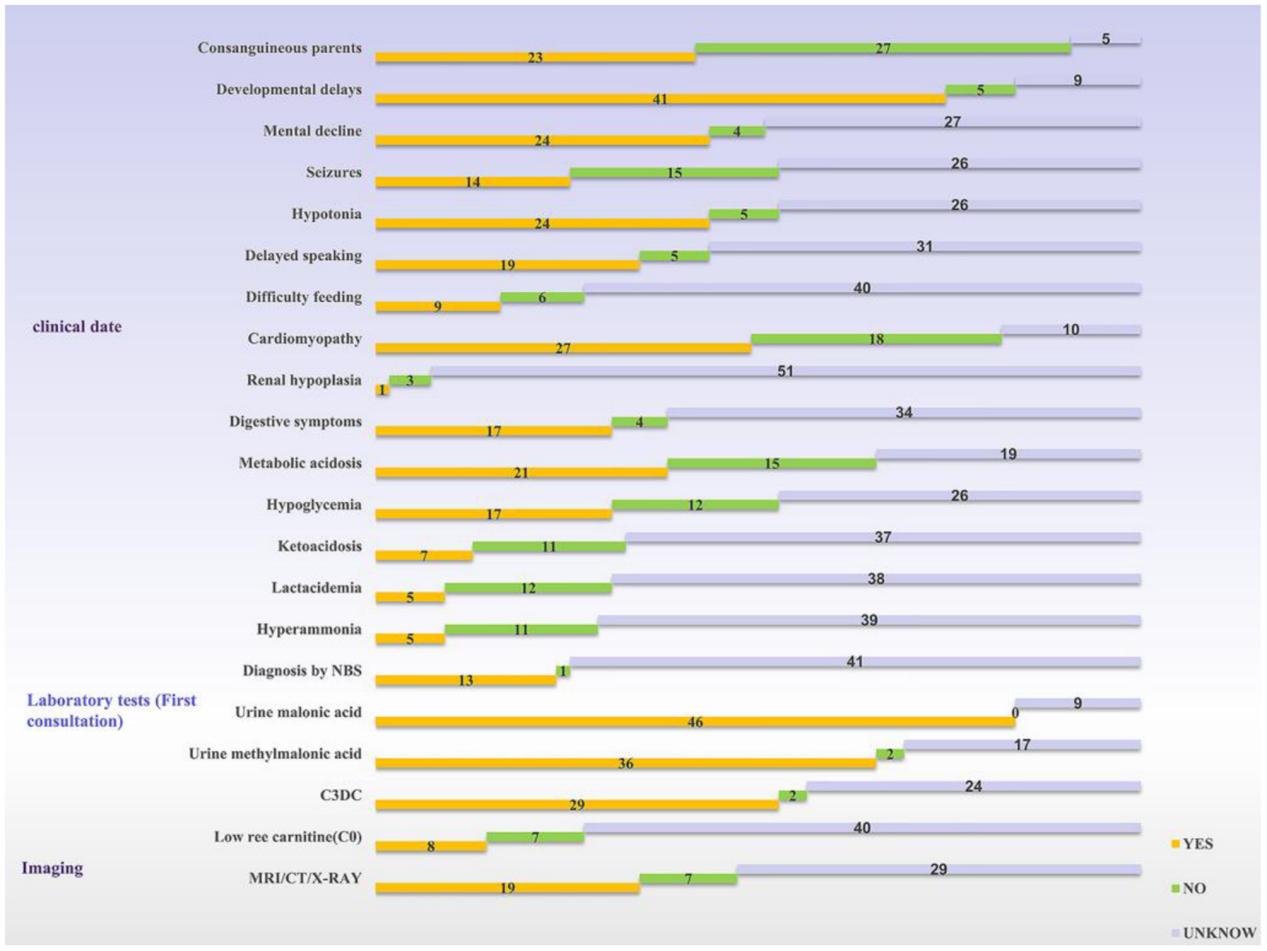
Statistics of clinical information reported in the literature. The yellow bar means the logo is present, the green bar means it is not, and the silver bar means it is not mentioned in the article. NBS, newborn screening; C3DC, malonylcarnitine.

According to the review, 54 patients have been described in the literature so far (3–32). Development retardation, cardiomyopathy, hypotonia, and hypothermia were the most common symptoms. Among all the cases reviewed in this study, there were twice as many male patients as female patients, and nearly 42% of families were consanguineous. Of these, developmental delay was the most common clinical sign (75%, 41/55), cardiomyopathy was seen in about half of the patients, and central nervous system disorders were also common; only one patient was found being described with renal hypoplasia. Urinary malonic acid is a key diagnostic marker for MCD deficiency and 84% of patients demonstrated a high level of malonic acid. Thirteen cases were diagnosed through newborn screening programs; the way of diagnosis for other cases was not mentioned (Figure 3).

**Figure 4:**
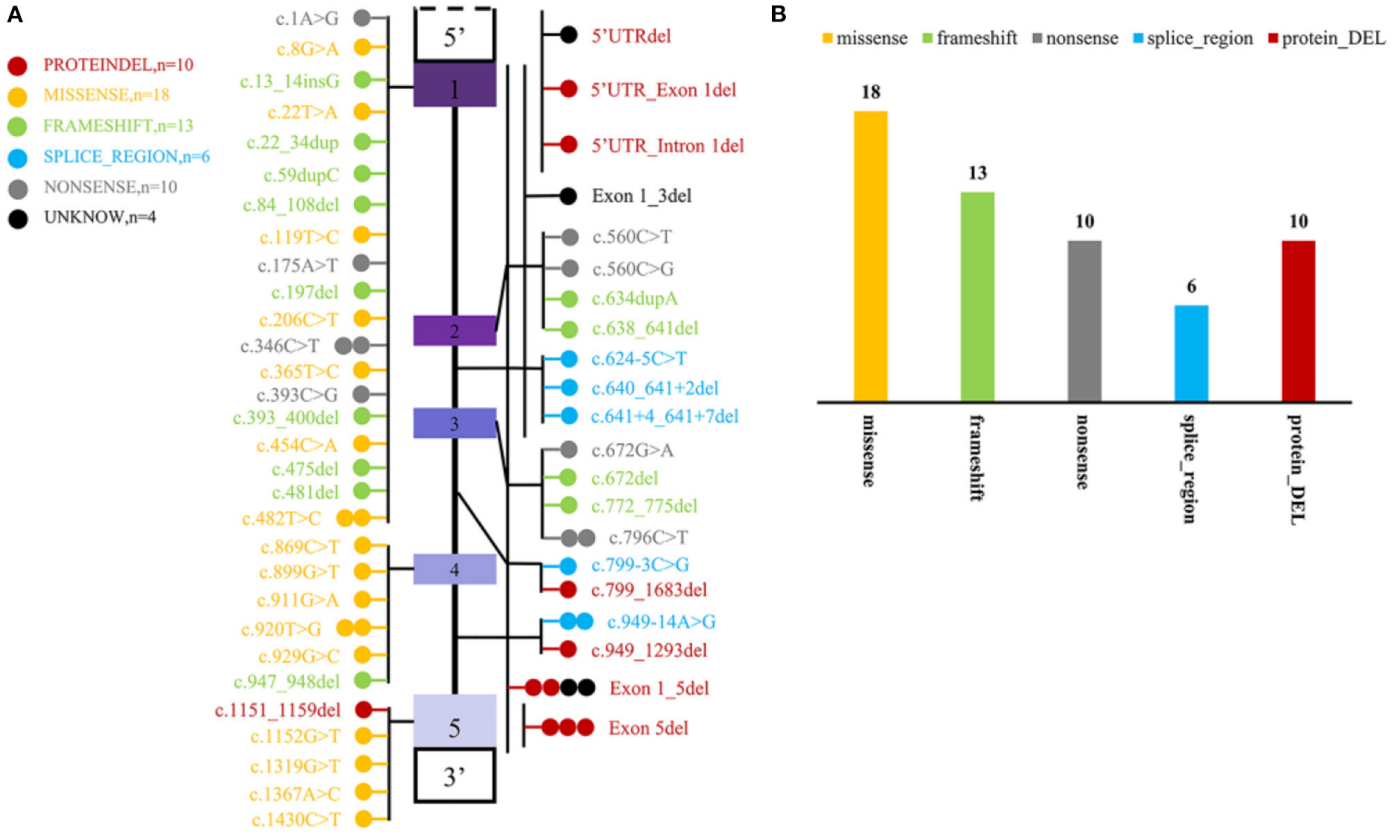
Statistics of mutation information of MYLCD gene reported in the literature. **(A)** Summary of MYLCD gene mutation loci described in the literature. The upper and lower blank boxes represent the regulatory regions of the MLYCD gene, and the colored boxes represent each of the five exons, with the region between them being introns. Each of these colored circles represents a different variant, and one circle means that there is one case of the mutant form represented by that circle. Twenty-nine variations are truncated variants, including non-sense, frameshift indels, and splicing variants, and 28 are missense variants or deletions without frameshift. The other four remaining variants are tentatively unclear and not found explicitly in the article; these four are indicated by black circles. **(B)** Statistical plot of protein effects produced by mutant loci. The specific colors representing the variants are described in the figure notes and are the same as in **(A)**.

To date, including the two mutant loci in this newly reported case, a total of 51 mutant loci have been reported to be associated with this disease. The mutation profile includes 10 CNV, 34 SNP, and 13 INDEL. Most variants are localized to coding regions (*n* = 52) and seldom to regulatory regions (*n* = 3) (Figure 4).

## Discussion

The clinical manifestations of malonic aciduria involve multiple phenotypes, but the majority include impaired heart and skeletal muscles and central nervous system (3). Here, we reported a girl with typical clinical features of malonic aciduria, including developmental retardation, hypotonia, and cardiomyopathy. The patient harbored novel compound heterozygous mutations in *MLYCD* which consisted of a non-sense mutation in the coding region, c.672del (p.Trp224^*^), and a large fragment deletion mutation in the promoter region from 5'UTR to intron1. After receiving an adaptive diet and symptomatic treatment, the patient recovered to a near-normal level of development.

The impaired catalytic activity of MCD caused by loss-of-function mutation of the MLYCD gene contributes to disorders of malonic acid metabolism. An extensive review of published case reports suggested that MLYCD mutations are comprised of point mutations, indels, and copy number alterations, which were detected by molecular techniques such as DNA-NGS, MLPA, array, and PCR. While most of the mutations occurred in the coding regions, there were still a few exceptions. In our case, one private mutation located in the regulatory region far beyond the detection limit of whole exome sequencing led to the failure of the first round of WES sequencing. Therefore, we used WGS which allows the detection of exons, introns, and regulatory sequences simultaneously.

We summarized the genotypes and phenotypes of previous cases in Supplementary Table 2 in Supporting Information and found that the correlation between the genotypes and phenotypes of MCD deficiency is still a puzzle. The degree of severity and onset time of the disease varied even among the patients who had the same mutation (15, 24). The level of C3DC, an indicator of enzymatic activity of MCD, was less related to the severity of the disorders. There are some explanations for this discrepancy. First, besides the major gene, modifier genes may also exert their influence on monogenic inherited diseases as conveyed by genomic analysis. Second, epigenomic factors may play a role in the pathogenesis of inherited disorders. Thus, DNA-seq combined with RNA-seq could be a solution that can better discover the correlation between the genotypes and phenotypes of MCD deficiency. Newborn screening may be a powerful tool for rapid diagnosis of MCD deficiency, enabling a pre-clinical treatment before more prominent symptoms appeared. According to our summary of the literatures, only a small fraction of patients were screened neonatally, partly due to the rarity of MCD deficiency. Training clinicians to recognize the high-risk factor of inherited diseases is urgently needed.

Currently, the etiological treatment for MCD deficiency is unavailable, while diet is the mainstay of treatment. In this reported case, the patient was treated with a special diet, L-carnitine supplementation, digitalis, and ACEI drugs after diagnosis. Three months after treatment, the cardiomyopathy improved significantly, the heart size and cardiac function recovered gradually, and the motor development improved to almost normal.

In conclusion, novel heterozygous *MLYCD* mutations NM_012213.2: c.672delG and 5'-UTR-exon1-intron1 can reduce the catalytic activity of MCD, leading to MCD deficiency. MCD deficiency is extremely rare, with diverse clinical manifestations. Neonatal metabolic screenings detect the disease at an early stage and genetic tests confirm it. After the diagnosis of the disease, follow-up monitoring should be performed even in the absence of clinical symptoms, for the major organs (heart, brain, etc.) are more susceptible to the disease. Dietary modification and L-carnitine supplementation can significantly improve the prognosis of cardiomyopathy.

## Data availability statement

To protect the privacy of the patients, all data related to the patients cannot be made available for public access, but all sequencing results from this manuscript are secured at Jiangxi Provincial Children's Hospital and are available from the corresponding author (yeduanjk@163.com; icemade@hotmail.com) upon reasonable request and approval by the Ethics Review Committee.

## Ethics statement

The studies involving human participants were reviewed and approved by the Institutional Review Ethics Board of Jiangxi Provincial Children's Hospital. Written informed consent for molecular test and publication of this case report were obtained from the patient's parents.

## Author contributions

FaX and JH collected the clinical information and peripheral blood samples. YZ helped to collect the clinical samples. YW collected literature. FaX and YW wrote the original draft preparation. HL contributed to the genetic counseling. JD, FeX, and HL supervised the study. All authors have read and agreed to the published version of the manuscript.
