# Supplementary material for: Case report: A novel 5'-UTR-exon1-intron1 deletion in MLYCD in an IVF child with malonyl coenzyme A decarboxylase deficiency and literature review
Source: Front Med (Lausanne). 2023 May 3;10:1160879. doi: 10.3389/fmed.2023.1160879 (PMC10189016; doi:10.3389/fmed.2023.1160879)
Supplement: Supplementary file 1 [file Table_1.DOC]

**Supplementary data:**

Figure 1: Results of the patient's echocardiogram at the time of admission.


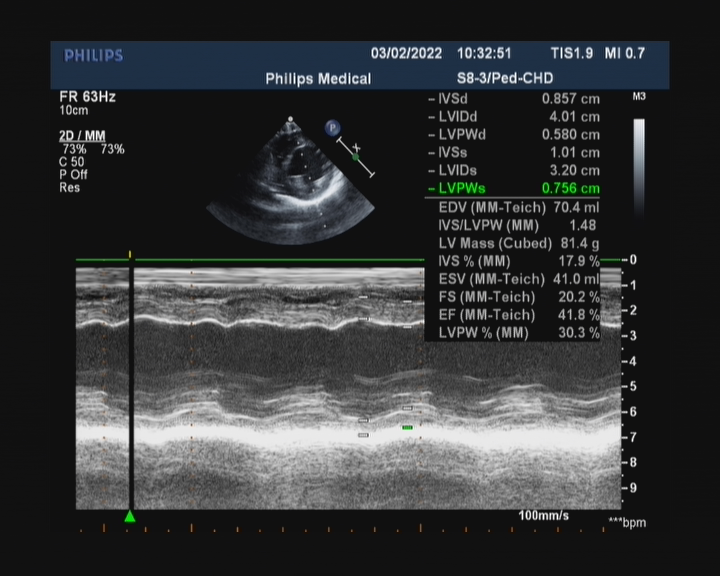


Table 1 Comparison of cardiac ultrasound measurements[[1]](#footnote-2)

|  | **LVDd（mm）** | **LVDs（mm）** | **EF（%）** | **LVFS（%）** | **IVSd（mm）** | **IVSs（mm）** |
| --- | --- | --- | --- | --- | --- | --- |
| **On admission** | 40 | 32 | 42 | 20 | 6 | 10 |
| **After 1 week of treatment** | 33 | 25 | 48 | 23 | 6 | 7 |
| **After 3 months of treatment** | 27.6 | 17.3 | 69.2 | 37.3 | 7.4 | 8.7 |
| **After 6 months of treatment** | 24.3 | 14.9 | 71.4 | 38.7 | 7.8 | 10.8 |
| **After 7 months of treatment** | 24.7 | 14.7 | 73.5 | 40.5 | 6.8 | 9.2 |

Table 2 The relationship between genotype and clinical phenotype[[2]](#footnote-3)

| case | Basic information of patients | | | | Genetic and protein changes | | Clinic date | | | | | | |
| --- | --- | --- | --- | --- | --- | --- | --- | --- | --- | --- | --- | --- | --- |
|  | Gender | Age of diagnosis | Country | Consanguineous parents | Mutation | Protein changes | Developmental delays | Central nervous system abnormalities | Cardiomyopathy | Digestive symptoms | Metabolic abnormalities | Renal hypoplasia | Brain imaging |
| 1 | M | 5y | Australia | Y | c.8G>A | p.G3D | + | / | / | + | + | / | / |
| 2 | M | 10m | Australia | N | c.475delG | Frameshift | + | + | / | + | + | - | - |
| 3 | M | 3.9y | Scotland | Y | c.949-14A>G | Generate new splicing site | + | + | / | + | / | / | / |
| 4 | F | 1y | Scotland | Y | c.442C>G + c.560C>T | p.S187X, no protein detected | + | + | + | + | + | / | - |
| 5 | M | 7m | Spain | N | c.119T>C | p.M40T | + | + | + | / | / | / | / |
|  | MA002 | / | / | / | c.947_948del | Truncated proteins | / | / | / | / | / | / | / |
|  | / | / | / | / | c.624-5C>T | Low expression | / | / | / | / | / | / | / |
| 6 | M | 4d | Lebanon | Y | c.393C>G | p.Y131X | + | + | - | + | + | / | + |
| 7 | M | 4y | Spain | N | c.84-108del | Frameshift | + | + | + | + | + | / | / |
| 8 | M | 10mdead | America | N | c.638–641del | Frameshift | + | + | + | / | + | + | / |
| 9 | F | 6m | Germany | N | c.59dupC + c.634dupA | Frameshift | / | + | + | / | + | / | / |
| 10 | F | 7m | Germany | N | c.59dupC + c.634dupA | Frameshift | / | - | + | / | - | / | / |
| 11 | M | 12y | Türkiye | Y | c.1151_1159del | p.W384_Q386del | + | + | / | + | / | / | + |
| 12 | F | 21m | Spain | N | c.206C>T + ? | p.A69V + [?] | + | + | - | / | + | - | + |
| 13 | M | 6d | / | N | c.482T>C + ? | p .L161P + [?] | / | / | / | / | + | / | / |
| 14 | F | 2y | Morocco | Y | Homozygous mutations at regulatory sites | Protein deletion | + | + | - | / | + | / | + |
| 15 | M | 5d | Italy | N | c.772_775del | Frameshift | + | + | + | + | + | / | + |
| 16 | M | 13y | / | / | c.560C>G + [Complete deletion of genes] | p.Ser187X + p.0 | + | / | - | / | / | / | / |
| 17 | M | 30m | / | Y | c.796C>T | p.Gln266X | + | / | + | / | - | / | / |
| 18 | M | 10y | / | Y | c.796C>T | p.Gln266X | + | + | - | / | - | / | / |
| 19 | F | 5d | / | Y | c.481delC | p.Leu161CysfsX18 | / | / | + | / | + | / | + |
| 20 | M | 6m | / | / | 5'UTRdel | p.?，MLYCD5'deletion | + | + | + | / | + | / | / |
| 21 | M | 30m | / | / | c.1319G>T + c.1367A>C | p.Ser440Ile + p.Tyr456Ser | + | + | / | / | / | / | / |
| 22 | F | 10m | / | Y | c.949_14A>G | Frameshift | + | / | - | + | + | / | / |
| 23 | F | 6m | / | / | c.899G>T + c.1430C>T | p.Gly300Val + p.Ser477Phe | - | - | - | + | - | / | / |
| 24 | / | / | / | / | c.799-1683_949-1293del3128 | Protein deletion | / | / | / | / | / | / | / |
| 25 | F | 5d | Asian | Y | c.197del | p.Gly66ValfsX7 | + | - | + | / | / | / | / |
| 26 | M | 3y2m | China | N | c.920T>G | p.Leu307Arg | + | + | - | - | - | / | + |
| 27 | F | 10m | Caucasian | N | c.393_400del | Frameshift | - | - | + | - | - | - | / |
| 28 | M | At birth | Italy | N | c.672G>A + c.869C>T | p.W224X + p.S290F | + | + | / | / | / | / | - |
| 29 | F | 5y | Türkiye | Y | Heterozygous Exon 1_5del | / | / | + | - | / | - | / | / |
| 30 | M | 3y | Türkiye | Y | Heterozygous Exon 1_5del | / | / | + | - | / | - | / | / |
| 31 | F | At birth | Türkiye | Y | Homozygous Exon 1_5del | Protein deletion | + | + | - | / | - | / | / |
| 32 | F | At birth | Türkiye | Y | Homozygous Exon 1_5del | Protein deletion | / | / | + | / | - | / | / |
| 33 | F | 6m | Finland | N | c.22T>A + c.454C>A | p.M1K + p.H152N | + | + | / | / | + | / | + |
| 34 | F | 9y7m | China | N | Exon1_3 157del+c.911G>A | p.G304E | - | + | - | / | - | / | - |
| 35 | M | 1y | / | Y | c.13_14insG | p. P6Afs^*^202 | + | / | + | / | - | / | + |
| 36 | M | 8d | Caucasian | N | 5'UTR_Exon 1del | / | + | + | + | / | + | / | + |
| 37 | M | 8d | Mexico | N | c.640_641+2del | p.Glu214Glyfs^*^2 | + | + | - | / | / | / | - |
| 38 | F | 6d | Mexico | N | Exon 5del | / | + | + | + | / | / | / | + |
| 39 | M | 8d | Caucasian | N | c.365T>C + c.1152G>T | p.Leu122Pro + p.Trp384Cys | - | + | - | / | + | / | + |
| 40 | M | 6m | Mexico | N | Exon 5del | / | + | + | + | / | + | / | + |
| 41 | M | 21d | Mexico | N | c.641+4_641+7del | Generate new splicing site | + | + | + | / | / | / | + |
| 42 | M | 5d | Nigeria | N | c.22_34dup + c.799-3C>G | p.Arg12Leufs^*^200,missing spliceing site | - | + | + | / | / | / | / |
| 43 | F | 5d | Middle East | Y | c.929G>C | p.Arg31Pro | + | - | - | / | / | / | / |
| 44 | M | 17d | Mexico | N | Exon 5del | / | + | + | + | / | / | / | - |
| 45 | M | 3m | Korea | N | c.1A>G + Exon 1_3del | / | + | + | + | / | + | / | - |
| 46 | M | 14m | Türkiye | Y | c.175A>T + (Gene SIX6c.422A>G) | p.Lys59Ter + (p.His141Arg) | + | + | + | + | / | / | / |
| 47 | M | 2y | / | Y | c.346C>T | p.Gln116^*^ | + | / | + | + | + | / | + |
| 48 | M | 5y | / | Y | c.346C>T | p.Gln116^*^ | + | + | - | + | - | / | / |
| 49 | M | 8m13d | China | N | c.482T>C + Exon 2&Exon 5del | p.L161P | + | + | + | - | - | / | + |
| 50 | F | 3d | China | N | c.672del + 5'UTR_Intron 1del | p.Trp224^*^ + ? | + | + | + | - | / | / | + |

1. LVDd: left ventricular end diastolic dimension; LVDs:left ventricular end systolic diameter; EF: ejection fraction; LVFS: left ventricular fractional shortening; IVSd: interventricular septal thickness at diastole; IVSs: interventricular septal thickness at systolic [↑](#footnote-ref-2)
2. "+"：the sign presence；"-"：the sign obsence；"/"：not mentioned in the articl [↑](#footnote-ref-3)
